# Synthetic Spatial Foraging With Active Inference in a Geocaching Task

**DOI:** 10.3389/fnins.2022.802396

**Published:** 2022-02-08

**Authors:** Victorita Neacsu, Laura Convertino, Karl J. Friston

**Affiliations:** ^1^Wellcome Centre for Human Neuroimaging, Institute of Neurology, University College London, London, United Kingdom; ^2^School of Life and Medical Sciences, Institute of Cognitive Neuroscience, University College London, London, United Kingdom

**Keywords:** active inference, spatial foraging, uncertainty, goal-directed behavior, geocaching, navigation, free energy principle

## Abstract

Humans are highly proficient in learning about the environments in which they operate. They form flexible spatial representations of their surroundings that can be leveraged with ease during spatial foraging and navigation. To capture these abilities, we present a deep Active Inference model of goal-directed behavior, and the accompanying belief updating. Active Inference rests upon optimizing Bayesian beliefs to maximize model evidence or marginal likelihood. Bayesian beliefs are probability distributions over the causes of observable outcomes. These causes include an agent’s actions, which enables one to treat planning as inference. We use simulations of a geocaching task to elucidate the belief updating—that underwrites spatial foraging—and the associated behavioral and neurophysiological responses. In a geocaching task, the aim is to find hidden objects in the environment using spatial coordinates. Here, synthetic agents learn about the environment via inference and learning (e.g., learning about the likelihoods of outcomes given latent states) to reach a target location, and then forage locally to discover the hidden object that offers clues for the next location.

## Introduction

Foraging is a type of goal-directed search process whereby (biological or synthetic) agents explore a given space with the purpose of discovering resources of (sometimes) limited availability. This search process is encountered in literature under various frameworks such as navigation (Montague et al., 1995; Rutledge et al., 2009; Humphries and Prescott, 2010; Pearson et al., 2014; Constantino and Daw, 2015; Kaplan and Friston, 2018), attention and visual salience (Itti and Koch, 2000; Parkhurst et al., 2002), or semantic memory (Hills et al., 2012; Todd and Hills, 2020). Each of these frameworks considers different components of complex multi-network and multi-function behavior. Successful foraging in certain animals engages the prefrontal cortex (Jung et al., 1998), decision making and reward circuits—such as the dorsal anterior cingulate cortex (Calhoun and Hayden, 2015) and the basal ganglia—as well as hippocampal and para-hippocampal areas involved in spatial navigation (Seamans et al., 1998; Kolling et al., 2012; Barry and Burgess, 2014), and planning. The basic need for food—to maintain homeostasis—also played a major role in the progressive evolution of cognitive structures (and functions) that would subsequently find a place within the core of abstract thinking in humans and primates. Indeed, accounts of sentient behavior must consider not only the specific task-dependent goal, but also uncertainty about the environment in which the agent acts. Context-dependent behavior applies not only to the physical space, but also to the abstract context of thoughts and decision making.

Foraging is a fundamental skill for survival. The ability to find food in the environment is conserved across species, although this is expressed in different ways depending on the specific species—and can take particularly abstract and complex forms in humans. This species-dependent characteristic of foraging becomes immediately evident if we consider the sub-processes involved, most of which are usually attributed to the prefrontal cortex in humans and primates (Rudebeck and Izquierdo, 2021). These sub-processes include evaluation (such as value-based decision making), prediction, and action (such as learning about uncertainty, action selection, patch-leaving problems, and matching) and social cognition (Rudebeck and Izquierdo, 2021). In recent years, complementary work in neuroscience (especially in the field of human and primate decision making) and ethology has appealed to a more holistic understanding of decision making in light of core (information) foraging processes. Simultaneously, the evolution of foraging-related structures across species becomes necessary for building the decision-making skills observed in humans (Mobbs et al., 2018). For the purpose of the current work, we focus on one of these important aspects, namely on uncertainty reduction via exploration. Uncertainty proved to have a non-trivial role in foraging (Anselme and Güntürkün, 2019). Indeed, higher level of uncertainty in foraging and goal-oriented tasks boosts exploratory behavior and foraging motivation in both animals and humans. This “boost” is reflected in an increased dopaminergic response from the mid-brain, in particular the nucleus accumbens (Le Heron et al., 2020).

The role of uncertainty in cognition has been investigated under different assumptions and frameworks (Grupe and Nitschke, 2013; Hasson, 2017; Peters et al., 2017; Mukherjee et al., 2021; Walker et al., 2021). In learning processes, uncertainty has a direct relationship with statistical and parametric learning, in that the latter aims to find patterns of consistent associations over separate experiences, while the modulation of the former directly affects the latter via predictive processes (Hasson, 2017). When it comes to action, the effect of uncertainty underwrites epistemic behavior (namely, information gathering). Within active inference, uncertainty can arise at a number of levels: from the ambiguity associated with imprecise (likelihood) mappings from environmental states to sensory observations to the uncertainty that accrues through (prior) probabilistic state transitions (Parr and Friston, 2017).

Within this framework, two main kinds of uncertainty have been considered. On one hand, *epistemic uncertainty* can be related the temporal dynamics of the environment, in terms of uncertain transitions across states—transitions that could produce uncertainty reducing outcomes. On the other hand, *aleatoric uncertainty* can also result from the statistical mapping from hidden states to sensory observations, in terms of ambiguity in the sensory signal (Hüllermeier and Waegeman, 2021). Generally speaking, in uncertainty quantification, epistemic uncertainty reflects what we don’t know and is reducible, while aleatoric uncertainty can only be reduced by sampling or measuring something more precisely. In our computational work, both of these types of uncertainty are in play. We will explain the theoretical basis of active inference in the next section. However, it is immediately evident that uncertainty plays a core role not only in action selection, but also in the epistemic behavior that results in updates of beliefs about external states of the world. We focus on the specific role of uncertainty in spatial foraging to elucidate, both theoretically and neurophysiologically, how goal-directed epistemic behavior depends on the level of uncertainty about internal representations of the state of the world—and the planned exchange with that world.

For completeness, we note that the field of foraging studies has benefited from a variety of approaches and disciplines, from neuroscience of decision-making and economics (Hayden, 2018; Mobbs et al., 2018) to computational neuroscience (Ward et al., 2000; Gheorghe et al., 2001; Davidson and El Hady, 2019), from ethology (Stephens, 2008) to social studies (Gabay and Apps, 2020), with the substantial contribution of memory and spatial navigation research (Gutiérrez and Cabrera, 2015; Kerster et al., 2016; Nauta et al., 2020). For a thorough perspective on the topic, please refer to relevant reviews (Hayden and Walton, 2014; Hall-McMaster and Luyckx, 2019; Gabay and Apps, 2020). Although each of these approaches has shaped an ecologically valid and holistic understanding of foraging, the field lacks a unifying framework that can bridge between conceptual propositions and higher-scale (formal) theories and, crucially, neurophysiological mechanisms. The active inference framework is a promising candidate to achieve this. Not only does it provide a first principles conceptual account of real-world behavior and cognition, but it also features an accompanying neuronal process theory. This allows us to simulate both real-world behavior and the underlying neurophysiological mechanisms in a way that is biologically plausible.

Here, we use a geocaching task to build a generative model of foraging. With our model, we aim to show how both epistemic (explorative) and reward-seeking (exploitative) behaviors arise from the same generative model of the world. We focus on one of the core aspects of foraging—uncertainty reduction—as contextualizing spatial exploration and action selection. We succeeded in reproducing a simplified naturalistic behavior using a goal-directed task. Moreover, we report a set of neurophysiological simulations, which confirm the biological plausibility of the model and the role of dopamine in foraging and uncertainty reduction, as shown in previous studies (Fiorillo et al., 2003; Niv et al., 2005; Friston et al., 2014; Li et al., 2016; Gershman, 2017; Jo et al., 2018; Le Heron et al., 2020). Similarly to our proposal, recent work (Schwartenbeck et al., 2019) developed a consistent active inference account of goal-directed exploration, which provides complementary insights on the pivotal role of exploration-exploitation balance in a T-maze task with risk options. Here, we extend on this foundation toward a generalization of the theory in foraging behavior in the environment, where the binary decision-making choice is substituted by multidirectional goal-directed navigation. As we will show, the same principles succeed in reproducing spatial foraging behavior in an open environment.

In what follows, we summarize active inference with its implicit minimization of variational and expected free energy, offering a brief account of planning as inference (Botvinick and Toussaint, 2012; Friston et al., 2017a). We then describe the generative model used for numerical analyses. The subsequent section presents a series of illustrative simulations showcasing planning and foraging behavior, their underlying belief updating, and prospective neurophysiological correlates. In the final section, we review the numerical experiments in light of current empirical findings in the spatial foraging literature.

## Active Inference Basics

The basic notion underlying active inference is that biological organisms are inference machines that minimize (variational) free energy or, equivalently, maximize model evidence. This can also be defined as minimizing uncertainty about the environment (Friston et al., 2017a), or self-evidencing (Hohwy, 2016). Active inference rests on generative models of the environment in the form of beliefs about contingencies in the experienced world. Generative models can be thought of as alternative hypotheses about the unobservable causes that generate the agent’s observations. Each generative model is specified as a joint probability distribution over policies (i.e., sequences of actions or plans), hidden states, and outcomes. In brief, the agent uses sensory data (i.e., observations) to update its beliefs about hidden (i.e., unobservable) states and the most likely policies (i.e., sequences of actions) it should pursue, a process known as *inference*. Furthermore, active inference agents hold and optimize beliefs about their behavior. They select actions from posterior beliefs about policies (i.e., plans), which solicit new observations, in line with the goal of fulfilling prior preferences and resolving uncertainty. Perception and action are therefore optimized simultaneously: perception involves optimizing posterior beliefs about hidden states, whereas action involves the optimization of beliefs about policies (i.e., planning). This inference rests upon beliefs about model parameters encoding various contingencies that are themselves optimized over time through learning. Typically, inference proceeds moment to moment, while learning is a slower process under the prior assumption that states of affairs change more quickly than the context or contingency that is encoded by parameters. *Parametric learning* entails the optimization of beliefs about relationships implicit in the interaction between different (latent) variables in the environment, where actions are selected to resolve uncertainty about hidden states *and the parameters of a generative model*. Epistemic foraging to resolve uncertainty about latent states and parameters is often described in terms of salience and novelty, respectively (Parr and Friston, 2019; Schwartenbeck et al., 2019). Model parameters can encode beliefs (usually as concentration parameters) about likelihoods (of outcomes given hidden states), transitions (among states), preferences (for outcomes), initial states, and policies, typically designated by the matrices **A**, **B**, **C**, **D**, and **E**, respectively.

The (variational) *inference* process in Active Inference can therefore be seen as optimizing posterior beliefs about the causes of sensorial experience for past, present, and future (latent) states, based on observations, and contingent upon the pursuit of specific policies (Friston et al., 2017a). In what follows, we briefly outline inference, policy selection and learning in terms of belief updating as a minimization of variational and expected free energy.

First, we cast the process of inference as the minimization of variational free energy—also known as an evidence bound (Winn and Bishop, 2005)—with regards to the sufficient statistics of an approximate posterior distribution over the hidden causes *x* (representing hidden states *s*, and policies, π):


$$Q\,\left(x\right)=\arg\min_{Q\,\left(x\right)}F\approx P\,\left(x|\tilde{o}\right)\;\left(1\right)\,\mathrm{Variational}\,\mathrm{Free}\,\mathrm{Energy}$$

$$F=E_{Q}\,\left[\ln Q\,\left(x\right)-\ln P\,\left(\tilde{o}|x\right)-\ln P\,\left(x\right)\right]$$

$$=E_{Q}\,\left[\ln Q\,\left(x\right)-\ln P\,\left(x|\tilde{o}\right)-\ln P\,\left(\tilde{o}\right)\right]$$

$$=\underset{r\,e\,l\,a\,t\,i\,v\,e\,e\,n\,t\,r\,o\,p\,y}{\underbrace{D_{K\,L}\left[Q\left(x\right)||P\left(x|\tilde{o}\right)\right]}}-\underset{\log e\,v\,i\,d\,e\,n\,c\,e}{\underbrace{\ln P\,\left(\tilde{o}\right)}}$$

$$=\underset{c\,o\,m\,p\,l\,e\,x\,i\,t\,y}{\underbrace{D_{K\,L}\left[Q\left(x\right)||P\left(x\right)\right]}}-\underset{a\,c\,c\,u\,r\,a\,c\,y}{\underbrace{E_{Q}\,\left[\ln P\,\left(\tilde{o}|x\right)\right]}}$$

Where $\tilde{o}=\left(o_{1},\ldots ,o_{t}\right)$ denotes observed outcomes up until the current time. This equation can be thought of as *perception*. It shows that minimizing variational free energy brings the agent’s Bayesian beliefs close to the true posterior beliefs by minimizing the relative entropy term (a term that is never less than zero). This is equivalent to forming beliefs about hidden states that provide an accurate but parsimonious—complexity minimizing—explanation for observed outcomes. Complexity is simply the difference between posterior and prior beliefs, i.e., the degree to which one “changes one’s mind” when updating prior to posterior beliefs.

Action and planning are usually expressed as selecting the action from the most plausible set of actions (i.e., policies) that has the least expected free energy:


$$\pi^{*}=\arg\min_{\pi }=\sum_{\tau }G\,\left(\pi ,\tau \right)\;\left(2\right)\,\mathrm{Expected}\,\mathrm{Free}\,\mathrm{Energy}$$

G⁢(π,τ)=EQ~⁢[ln⁡Q⁢(A,sτ|π)-ln⁡P⁢(A,sτ,oτ|o~,π)]=EQ~⁢[ln⁡Q⁢(A)-ln⁡Q⁢(A|sτ,oτ,π)]⏟(Negative)⁢novelty+E[lnQ(oτ|π)-lnQ(oτ|sτ,π)]Q~⏟(Negative)⁢salience-EQ~⁢[ln⁡P⁢(oτ)]⏟Extrinsic⁢value

Where $\tilde{Q}=Q\,\left(o_{\tau },s_{\tau }|\pi \right)=P\,\left(o_{\tau }|s_{\tau }\right)\,Q\,\left(s_{\tau }|\pi \right)$. The purpose of this expression is to identify the best sequence of actions (i.e., policy) and implicit action for the next time step. Note that this kind of planning—based on expected free energy—implies averaging the free energy expected following a policy under the predicted outcomes. The expected log evidence therefore becomes extrinsic value: the extent to which outcomes conform to prior preferences. In economics, this term is known as utility (Fishburn, 1970), and in behavioral psychology, it corresponds to reward (Barto et al., 2013; Cox and Witten, 2019). Likewise, the expected relative entropy becomes the information gain pertaining to unknown model parameters (i.e., novelty) and unknown hidden states (i.e., salience). These measures are sometimes referred to as intrinsic or epistemic values and form the basis of artificial curiosity (Schmidhuber, 2006; Ngo et al., 2012; Schillaci et al., 2020). They quantify the value of the evidence accumulated if agents were to pursue a particular plan. Maximizing these intrinsic values can be seen as a form of optimal information gain or active learning (MacKay, 1992; Oudeyer and Baranes, 2008; Baranes and Oudeyer, 2013), where curiosity resolves uncertainty about states of the world and their contingencies—in accord with the principles of optimum Bayesian experimental design (Lindley, 1956).

Whereas salience is associated with beliefs about the current state of affairs in the world, and how they will unfold in the future, novelty is the reducible (epistemic) uncertainty about the probabilistic contingencies themselves, and the causal structure they entail (i.e., the causal structure of the environment). In other words, novelty affords the opportunity to resolve uncertainty about what would happen if agents engaged in a specific course of action (i.e., “what would happen if I did this”). An alternative way of decomposing expected free energy is into expected (in)accuracy and complexity—that can be understood as ambiguity and risk; namely, the (aleatoric) uncertainty that pertains to ambiguous outcomes and the risk that actions will bring about outcomes that diverge from prior preferences. This means that minimizing expected free energy resolves both epistemic and aleatoric uncertainty.

Parametric learning optimizes the parameters of the (generative) model. Active Inference agents with discrete state space generative models usually have priors (e.g., **A**, **B**, etc.) and hyper-priors (e.g., *a*,*b*, etc.) that encode beliefs about model parameters (Friston et al., 2016). Given that parametric beliefs (e.g., **A**) are defined as categorical distributions, an appropriate hyper-prior encoding the mapping between relevant couplings (e.g., state-outcome) is specified in terms of Dirichlet concentration parameters. Given a state (*s*), the belief about the probability of an outcome is:


(3) $$\begin{array}{c} P\,\left(o|s,A\right)=C\,a\,t\,\left(A\right)\\ P\,\left(\text{A}|a\right)=D\,i\,r\,\left(a\right)\Rightarrow \left\{ \begin{array}{c} E_{P\,\left(A|a\right)}\,\left[{\text{A}}_{i\,j}\right]=\frac{a_{i\,j}}{\sum_{k}a_{k\,j}}\\ E_{P\,\left(A|a\right)}\,\left[\ln{\text{A}}_{i\,j}\right]=\psi \,\left(a_{i\,j}\right)-\psi \,\left(\sum_{k}a_{k\,j}\right) \end{array}\right. \end{array}$$

Where ψ is the digamma function. Agents then accrue Dirichlet parameters during exposure to new observations, permitting them to learn. Updates over these parameters involve the accumulation of Dirichlet parameters that represent the mapping from hidden states to the observed outcome (Friston et al., 2016; Da Costa et al., 2020). For instance, updates to the concentration parameters of the likelihood mapping are expressed as:


(4) $$\text{a}=a+\sum_{\tau }{\text{s}}_{\tau }⊗o_{\tau }$$

Where *a* and **a** represent prior and posterior concentrations parameters, respectively, and **s**_τ_ denotes posterior expectations about the hidden states.

Given that accumulating (Dirichlet) concentration parameters (in this case over the likelihood matrix) is equivalent to the type of change seen in activity or experience-dependent plasticity (Brown et al., 2009; Friston et al., 2017a), it can be regarded as a synaptic strengthening each time neurons encoding states and observations (coupled by that synapse) are active simultaneously. This formulation therefore provides a mathematical description of Hebbian or associative plasticity.

In active inference, extrinsic (utility) and intrinsic (epistemic) values are optimized in tandem, since policy selection is underwritten by expected free energy, which in itself entails a dual pursuit: maximizing utility and information gain (Friston et al., 2017b). Normally, the behavior of Active Inference agents is dominated by epistemic incentives until uncertainty about the environment has been resolved. Thereafter, extrinsic incentives take charge, giving rein to exploitative behavior. For a detailed account of Active Inference and associated tenets, please see Friston et al. (2016; 2017a,c;^2018^), Da Costa et al. (2020), and Smith et al. (2020). In the current work, we call upon these intrinsic and extrinsic values to simulate information and goal seeking behavior in a novel environment.

## The Generative Model and Belief Propagation

In summary, generative models are joint probability distributions over observed outcomes, latent causes, and sequences of actions (i.e., policies), necessary to optimize beliefs and subsequent behavior. The active side of the inference process corresponds to inverting a generative model using observed outcomes (i.e., generating consequences from causes), and forming posterior expectations about the hidden states (i.e., recovering causes from consequences). Crucially, in active inference these expectations include the most likely action, hence active inference. In this section, we describe the specific generative model used to simulate purposeful behavior and associated belief updating, and the slower accrual of evidence (i.e., associative plasticity). These distinct processes are emergent aspects of minimizing the variational bound on (negative log) model evidence described above. These processes have a reasonable degree of biological plausibility, enabling us to simulate neuronal responses and changes in synaptic efficacy during inference and learning, respectively (Friston et al., 2017a,c).

When using Active Inference schemes, the principal challenge lies in specifying a suitable generative model to capture the behavior and cognition induced by the task or problem in question, rather than devising a scheme for Bayesian optimal behavior. Once the generative model has been specified, model inversion (i.e., inference and learning) can use standard belief updating schemes (e.g., spm_MDP_VB_X.m, available in SPM12).^1^ The generative model we use in the following simulations is a deep temporal model (Friston et al., 2017d) based on a partially observable Markov decision process (POMDP). Under these sorts of models, there are generally four types of latent causes: *hidden states* (of the world) that generate observable outcomes, *policies* (i.e., sequences of actions being pursued) that specify transitions among the hidden states, *precision* encoding confidence in beliefs about policies, and *parameters* (e.g., likelihood).

**FIGURE 1:**
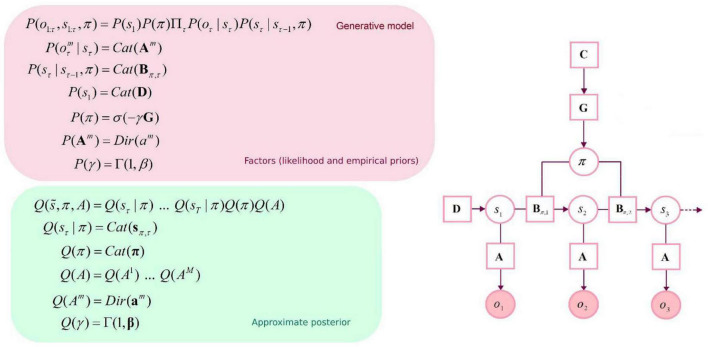
Graphical depiction of the generative model and approximate posterior. This discrete-state space temporal model has one hidden state factor: *location*. This factor generates outcomes in two outcome modalities: *where* and *what* (with two levels: reward or null). The likelihood A is a matrix whose elements are the probability of an outcome under every combination of hidden states. B represents probabilistic transitions among hidden states. Prior preferences over outcome modalities for each hidden state factor are denoted by C. The vector D specifies priors over initial states. *Cat* denotes a categorical probability distribution. *Dir* denotes a Dirichlet distribution (the conjugate prior of the *Cat* distribution). An approximate posterior distribution is needed to invert the model in variational Bayes (i.e., estimating hidden states and other variables that cause observable outcomes). This formulation uses a mean-field approximation for posterior beliefs at different time points, for different policies and parameters. Bold variables represent expectations about hidden states (in italic). Transparent circles represent random variables, and shaded circles denote observable outcomes. Squares denote model parameters and expected free energy.

The generative model is parameterized by a set of arrays (i.e., matrices and vectors): a likelihood matrix encoding probabilistic mappings from (hidden) state factors to outcome modalities (**A**), transition probabilities among the different hidden states given particular actions (**B**), prior preferences over outcome modalities for each hidden state factor (**C**), and finally, priors over initial states (**D**). As mentioned above, these matrices are parameterized with Dirichlet (concentration) parameters that accumulate during experience: the amalgamation of a given hidden state and outcome effectively adds a concentration parameter (i.e., a count) to the appropriate element of the likelihood mapping. Here, there are two outcome modalities: the first (*what*) registers rewarding outcomes with two levels (*reward* vs. *null*). The second modality reports the current location in the space being explored (*where*). Outcomes are generated from a single hidden state factor (*location*), corresponding to locations in a 10×10 grid. Please see Figure 1 for a graphical depiction of the generative model. There are 5 allowable actions: up, down, left, right, and stay. These actions induce 5 transition matrices that play the role of empirical priors. The outcomes *reward: present* and *reward: null* were assigned a utility (i.e., relative log probability) of 3 and 0, respectively. With these utility values, the synthetic agent would “prefer” (i.e., expect) a *reward: present* outcome about 20 times more than the *reward: null* outcome. The agent also prefers being in proximity of the target location (i.e., *reward: present*). In summary, we specified a minimal generative model necessary to illustrate navigation and (epistemic) foraging in which the causes of observable outcomes were locations in space. The observations available to an agent comprised two sorts. The first told it unambiguously where it was and the second described what happens at each location, in terms of preferred or non-preferred outcomes. The agent can move around this space, taking one step at a time—knowing its location but not necessarily knowing location-specific outcomes in the reward modality.

**FIGURE 2:**
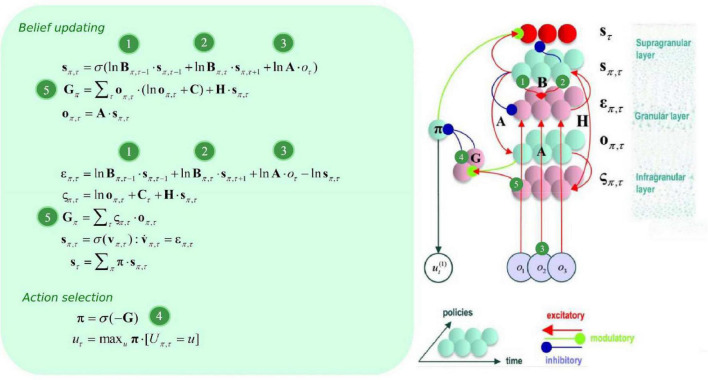
Belief update and propagation. The left panel shows the equations that underlie (approximate Bayesian) *inference* and action selection. The differential equations (middle left) can be construed as a gradient descent on (variational) free energy, and are defined in terms of prediction errors. Policy expectations are computed by combining the two types of prediction error (*state* and *outcome*), via a softmax function (message 4). State PE quantifies the difference between expected states for each policy (messages 1, 2, and 3), whereas outcome PE computes the difference between expected and predicted outcomes, and is weighted by the expected outcomes to estimate the expected free energy (message 5). The right panel displays the message passing implied by the belief update equations in the left panel. Neural populations are represented by the colored spheres, which are organized to reproduce recognized intrinsic connectivity for cortical areas. Red and blue arrows are excitatory and inhibitory, respectively. Green arrows are modulatory. Red spheres indicate Bayesian model averages, pink spheres indicate both types of PE. Cyan spheres represent expectations about hidden states and future outcomes for each policy. Connection strengths represent generative model parameters.

**B** can be thought of as an empirical prior, since it depends upon actions, which themselves are determined by policies π (i.e., it depends upon a random variable). Policies are *a priori* more probable if they minimize expected free energy **G**, which is contingent upon prior preferences about outcomes **C**, and (aleatoric) uncertainty about outcomes under each state **H** (please see Figure 2). Update equations (that allow agents to minimize free energy) are derived from the generative model, with consideration for neurobiological constraints. Briefly speaking, expected hidden states are updated by means of belief propagation. In active inference, this is achieved using a gradient descent on (variational) free energy for each hidden variable.

The ensuing solutions implement message passing from representations of the past (forward = message 1), future (backward = message 2), and observations (message 3) that update posterior beliefs over latent (hidden) states, allowing for both postdiction and prediction under each individual policy (see Figure 2). As new outcomes emerge, more likelihood messages contribute to the belief update, which makes for more informed posteriors. This recurrent message passing can be summarized as follows: the generative process (i.e., the environment) generates outcomes that update approximate posteriors about policies (i.e., plans), which are themselves contingent upon prior preferences and intrinsic value. The policies determine the selected action, and selected actions generate new outcomes.

To specify the gradient descent on (variational) free energy, we substitute *ln*⁡*s*_π,τ_ by *v*_π,τ_, and introduce a state prediction error (PE) variable ε_π,τ_ as described in Friston et al. (2017c):


(5) $$\begin{array}{c} {\text{s}}_{\pi ,\tau }=\sigma \,\left({\text{v}}_{\pi ,\tau }\right)\\ {\dot{\text{v}}}_{\pi ,\tau }=\varepsilon_{\pi ,\tau }\\ \varepsilon_{\pi ,\tau }=\ln{\text{B}}_{\pi ,\tau -1}\cdot {\text{s}}_{\pi ,\tau -1}+\ln{\text{B}}_{\pi ,\tau }\cdot {\text{s}}_{\pi ,\tau +1}+\\ \ln\text{A}\cdot {\text{o}}_{\tau }-\ln{\text{s}}_{\pi ,\tau } \end{array}$$

Which is formally equivalent to the differential equation in Friston et al. (2017a):


(6) $${\dot{\text{v}}}_{\pi ,\tau }=\varepsilon_{\pi ,\tau }=-\frac{\partial {\text{F}}_{\pi }}{\partial {\text{s}}_{\pi ,\tau }}$$

This basically says that we can understand neuronal dynamics as performing a gradient descent on variational free energy, or—more heuristically—minimizing various prediction errors.

Since this equation describes the rate of change of a log expectation (i.e., softmax of log expectations), a neurobiologically plausible interpretation is to associate the log expectations with the depolarization of neuronal populations, and the message passing itself (the softmax) with neuronal firing rates (Friston et al., 2017a). Please see Figure 2 for the ensuing belief update scheme. For an extensive description of belief update and their possible neurobiological mechanisms, please see (Friston et al., 2017a).

## Simulations and Results

Our numerical experiments focused on navigation and local foraging, respectively. In the navigation simulations, the agent sees a space comprising a 10 × 10 grid and navigates toward preferred target locations (specified with prior preferences over the location modality). For the foraging simulations, we zoom into a local area (also a 10 × 10 grid), where the agent engages in epistemic foraging to find a hidden object (i.e., rewarding location). After finding this object, the agent is given a new target location and the process repeats. The agent thus plans its trajectory toward its target location, and then explores the location to find hidden rewards. This object could be regarded as the cue that specifies the next target location. After navigating to the second target location, the agent again explores locally to find the hidden object. This process could continue ad infinitum. In this demonstration, both epistemic foraging and goal directed behavior are evinced via the minimization of (expected) free energy.

For the navigation phase, the agent starts at the entrance of the grid. Prior preferences prompt the agent to seek out target locations. Cues that directly inform the agent of its current location can be thought of as exteroceptive, whereas the observed outcomes (*reward* or *null*) can be thought of as interoceptive. The policy depth (i.e., planning horizon) involves four steps—that is, agents can evaluate distal (and possibly preferable) outcomes in the future, which allows them to plan and pursue the shortest trajectory toward the end goal (i.e., the first *rewarding* location). In this simulation there were 10 moves in total, enough to reach the target location using the shortest available path. Synthetic agents were endowed with prior knowledge about the environment—so that they were planning their trajectory in a familiar environment.

For the local foraging simulations, the agent has additional (i.e., epistemic) incentives in the form of uncertainty about the location that contains the *rewarding* outcome. In this context, agents explore the environment, initially motivated by curiosity about the parameters of the model (here, the likelihood matrices—see set of equations number 2). In other words, their behavior was driven by the novelty of the environment; namely, “what would happen if I went there?” To simulate exposure to this local novel environment, the prior Dirichlet parameters of the likelihood mapping (**A**)—encoding the mapping between hidden states and “*what*” outcomes (i.e., *reward* or *null* outcomes)—were set to a small value (i.e., 1/100). As a consequence, the expected free energy **G** acquires a non-trivial novelty term (Friston et al., 2017b). This phase of the simulations illustrates how agents learn about their environment by means of novelty-driven evidence accumulation. Technically, this entails the updating of Dirichlet parameters (encoding hidden state—outcome mappings) after 30 successive moves in the local environment. Once locations are visited, they lose their novelty (i.e., epistemic value), a process which endorses those policies that visit unexplored ground. Preferences for particular outcomes (i.e., *reward* and *location*) were formally the same as the prior preferences used in the navigation simulations. We also specified concentration parameters in the state transition matrix to simulate an additional type of learning—comparable to that of foraging in volatile environments—where (biological) agents have some degree of uncertainty about where exactly they will move to, based on where they have just foraged (and the actions they pursued).

**FIGURE 3:**
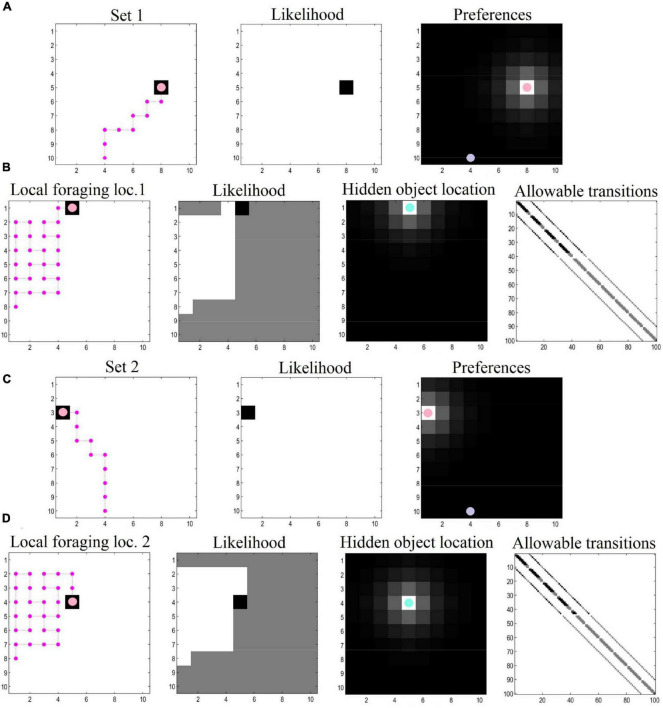
Navigation and local foraging behavioral results. **(A)** The agent plans and executes its (shortest available) trajectory toward the first target location, driven by prior preferences. The purple dot indicates the starting location. The agent has learned the likelihood mappings, which can be interpreted as having—and making use of—a map to reach the target location. **(B)** When the target location is reached, the agent explores the local area to find a hidden object, as it learns and discovers its environment. Here, the agent starts with a uniform distribution about the likelihood mappings, and has additional uncertainty pertaining to the transition matrix (i.e., uncertainty about where the agent finds itself given where it was previously and the action it has taken). This process involves a dual pursuit: discovering the environment and fulfilling a desire to find the hidden object. **(C,D)** After finding the hidden object, the agent receives a new target location and the process repeats (possibly ad infinitum).

Collectively, these simulations mimic the circumstances surrounding local foraging in geocaching, where agents freely explore the environment to discover a hidden object. The agent, however, maintains a dual imperative—to discover the environment by satisfying its curiosity, and at the same time, to realize prior preferences (i.e., of finding the object hidden in the environment). In Figure 3, we depict results of exemplar simulations for both types of simulations.

**FIGURE 4:**
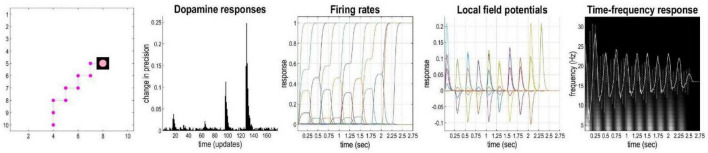
Simulated electrophysiological responses for a representative sequence of moves. The left panel shows the agent’s trajectory, followed by (synthetic) dopamine responses, firing rates, local field potentials and time-frequency responses. Please see main text for more details.

In active inference, as mentioned above, a softmax function is applied to (precision-weighted) expected free energy in order to optimize posterior beliefs about each policy. When new observations are available, the precision parameter is updated: the policy with the lowest (expected) free energy is more likely if the associated precision parameter is high (c.f., an inverse temperature parameter). The confidence that the inferred policy will produce preferred outcomes or resolve uncertainty about latent states is therefore represented by this precision parameter. Dopaminergic activity in the mid brain is thought to encode this type of precision (Schwartenbeck et al., 2014). Figure 4 illustrates representative simulated neural activity for the agent’s last planning and movement sequence (i.e., 10 movements) during the navigation simulations. In the current model, the phasic bursts observed in simulated dopaminergic responses (see Figure 4) indicate notable changes in precision at steps 1, 4, 6, and 8 (i.e., the 16th, 64th, 96th, and 128th iteration, respectively, in terms of updates—since there are 16 iterations of gradient descent per time-point). These suggest a change in confidence (i.e., the agent resolves uncertainty) about what policies to pursue, by eliminating other possible trajectories. In this scenario, at the first step, the agent eliminated the possibility of going right instead of up, an action that could equally have allowed it to reach the target using the minimum number of steps. At the 8th movement, the agent becomes confident about fulfilling its target location, and spends steps 9 and 10 within the *rewarding* state. This example shows how belief updating and decision making can be unpacked in terms of uncertainty and precision.

Firing rates indicate changes in beliefs over time about the state for each time-point. The fourth panel of Figure 4 depicts predicted local field potentials (depolarization), showing the rate of change in simulated firing rates for all (1,100) hidden state units (colored lines). This panel shows that visiting different locations evokes responses in different neuronal populations, and of variable degrees. Finally, the right panel displays neuronal responses associated with the *where* state beliefs before and after filtering at 4 Hz (dotted and solid line, respectively). These are superimposed upon a time-frequency decomposition of the averaged local field potential (over all simulated neurons). These show fluctuations in local field potentials at a theta rhythm that are phase-locked to induced responses over a wide range of frequencies (including gamma frequencies—not shown). This reproduces the characteristic theta-gamma coupling found in empirical studies of foraging and navigation in small animal studies (Bragin et al., 1995; Lisman and Redish, 2009; Buzsaki and Moser, 2013).

## Discussion

Learning about the environment is fundamental for human and animal behavior alike. In particular, foraging requires the interaction of several processes to maintain homeostasis, on which survival depends. Recent advances in both the neuroscience and ethology of foraging have emphasized the need for a holistic, ecologically valid approach. Although different disciplines contribute to the extensive body of work of foraging, in and across different species, the field lacks an integrative account. In this work, we address two key components of the complex process of foraging: uncertainty reduction and action selection. Our computational account of foraging succeeds in reproducing real-world behavior, and at the same time accounts for its neurophysiological correlates. The setup of our model, in its simplicity, tries to be ecologically valid and to account for the sequential nature of foraging; especially the accumulation of knowledge during epistemic foraging. Each outcome is indeed not only a partial goal of the task (a partial reward, if we will), but also the cue for the next outcome. In a similar way, when we explore the environment, we rarely have one-shot rewards but progressive cues that guide us closer and closer to the final location (for instance, an animal would first find a trace of its prey, the precise location of the prey, and so on, until it finally secures the final goal—and has to start again for the next meal).

Our results reproduce two levels of foraging behavior: goal-directed navigation (in the global environment) and epistemically-driven exploration (locally). In the first case, the goal is to follow a trajectory, given preferences for a target location. In the second this preference seeking motivation is contextualized by explorative or epistemic imperatives. Note that because the epistemic and preference parts of expected free energy are expressed as log probabilities, the policies selected can be viewed as reflecting the product of the probabilities *per se*. In other words, epistemic policies will be rejected if they have a very small probability of securing a preferred outcome. A very small probability of a preferred outcome corresponds to an aversive or surprising outcome, which means that prior preferences constrain the epistemic affordances of any behavior (under active inference).

Our simulations illustrate the effect of uncertainty on behavior and neuronal activity. This is particularly relevant in the second part of our simulations (local foraging). The degree of explorative behavior is modulated by the level of uncertainty about the state of the world. When uncertainty is high, action selection is built upon the explorative imperative of reducing uncertainty. The more the agent becomes confident about its surroundings (i.e., the more uncertainty is reduced), the more action selection is guided by exploitative behavior, when extrinsic gain is predominant, and less by exploration. Uncertainty reduction has, thus, a direct effect on action selection. As proposed in previous work, dopamine is responsible for encoding uncertainty over policies or decisions. In other words, the kind of beliefs—whose precision is modulated by dopamine—are beliefs about policies (sequences of actions, resulting in action selection). At a synaptic level, the modulation of precision could be thought of as neuromodulation or synaptic gain control (Parr and Friston, 2017). The firing rate of dopamine in the mid brain is nicely reproduced in our electrophysiological simulations. As expected, the agent becomes more and more confident about its predictions, which is reflected in a progressive increase in rates of beliefs updating and reduction of uncertainty.

The current work has some clear limitations. Although it succeeds in reproducing biologically plausible and real-world oriented foraging behavior, it does not account for several sub-processes involved in foraging. We prioritized clarity over complexity, and we did not develop our work with the purpose of including aspects of spatial navigation (such as the navigation system of hippocampal and para-hippocampal areas), patch-leaving problems, matching and social cognition. Another limitation of our work is the assumption that the model is given the target location as a fully formed prior preference. This could be interpreted as “information passing” of cues between individuals of the same group. However, a more extensive account of foraging would have to address how these prior location preferences were inferred or learned.

Although restricted in its focus, our model offers a preliminary account of foraging, both in terms of behavioral and neurophysiological responses. Future work could aim to extend this approach to include the missing elements of foraging. Active inference is indeed equipped to account for many aspects of sentient behavior, social behavior included. A successful extension of the model could also reproduce and investigate the neurophysiological role of other neurotransmitters in foraging. For example, the role of norepinephrine in setting the precision of state transitions—or the role of cholinergic neurotransmission in setting the precision of sensory or likelihood mappings (Doya, 2002, 2008; Parr and Friston, 2017). Moreover, active inference offers a promising approach to close the gap not only between behavior and neurophysiology, but also between foraging mechanisms across different species. Developmental and comparative neuroscience could benefit from *in silico*, evidence-informed modulation of model parameters to test different hypotheses about how foraging evolved over time—from simple living beings to more advanced primates and humans. This work offers one step toward a holistic conceptual and mechanistic understanding of foraging via a geocaching task in the active inference framework.

## Data Availability Statement

The original contributions presented in the study are included in the article/supplementary material, further inquiries can be directed to the corresponding author/s.

## Author Contributions

VN: conceptualization, formal analysis, and visualization. VN, LC, and KF: methodology and writing – review and editing. VN and LC: writing. All authors contributed substantially to conception, design, and writing of this article, and have read and agreed to the published version of the manuscript.

## Conflict of Interest

The authors declare that the research was conducted in the absence of any commercial or financial relationships that could be construed as a potential conflict of interest.

## Publisher’s Note

All claims expressed in this article are solely those of the authors and do not necessarily represent those of their affiliated organizations, or those of the publisher, the editors and the reviewers. Any product that may be evaluated in this article, or claim that may be made by its manufacturer, is not guaranteed or endorsed by the publisher.

## Footnotes

1
www.fil.ion.ucl.ac.uk/spm/software/download
